# Hormonal Regulation in Different Varieties of *Chenopodium quinoa* Willd. Exposed to Short Acute UV-B Irradiation

**DOI:** 10.3390/plants10050858

**Published:** 2021-04-23

**Authors:** Lorenzo Mariotti, Thais Huarancca Reyes, Jose Martin Ramos-Diaz, Kirsi Jouppila, Lorenzo Guglielminetti

**Affiliations:** 1 Department of Agriculture, Food and Environment, University of Pisa, via Mariscoglio 34, I-56124 Pisa, Italy; lorenzo.mariotti@unipi.it (L.M.); lorenzo.guglielminetti@unipi.it (L.G.); 2 Centro di Ricerche Agro-Ambientali “Enrico Avanzi”, University of Pisa, via Vecchia di Marina 6, I-56122 Pisa, Italy; 3 Department of Food and Nutrition, University of Helsinki, Agnes Sjöbergin katu 2, FI-00014 Helsinki, Finland; jose.ramosdiaz@helsinki.fi (J.M.R.-D.); kirsi.jouppila@helsinki.fi (K.J.); 4 Interdepartmental Research Center “Nutraceuticals and Food for Health”, University of Pisa, via del Borghetto 80, I-56124 Pisa, Italy

**Keywords:** indoleacetic acid, abscisic acid, chlorophyll fluorescence, flavonoids, jasmonic acid, salicylic acid, quinoa, ultraviolet-B

## Abstract

Increased ultraviolet-B (UV-B) due to global change can affect plant development and metabolism. Quinoa tolerates extreme conditions including high UV levels. However, the physiological mechanisms behind its abiotic stress tolerance are unclear, especially those related to UV-B. We previously demonstrated that 9.12 kJ m^−2^ d^−1^ may induce UV-B-specific signaling while 18.24 kJ m^−2^ d^−1^ promotes a UV-B-independent response. Here, we explored the effects of these UV-B doses on hormonal regulation linked to plant morphology and defense among diverse varieties. Changes in fluorescence parameters of photosystem II, flavonoids and hormones (indoleacetic acid (IAA), jasmonic acid (JA), abscisic acid (ABA) and salicylic acid (SA)) were surveyed under controlled conditions. Here, we showed that the sensitivity to short acute UV-B doses in varieties from different habitats is influenced by their parental lines and breeding time. UV-B sensitivity does not necessarily correlate with quinoa’s geographical distribution. The role of flavonoids in the UV-B response seems to be different depending on varieties. Moreover, we found that the extent of changes in JA and SA correlate with UV-B tolerance, while the increase of ABA was mainly related to UV-B stress.

## 1. Introduction

Light is an essential source of energy for photosynthesis, but also functions as a signal source about the environment, and thus regulates diverse morphological, physiological and developmental processes in plants. Moreover, multiple hormonal pathways are recruited and coordinated by light to mediate these light-regulated processes, resulting in a complex crosstalk between hormonal and light signaling pathways [1,2]. Ultraviolet-B (UV-B) radiation belongs to a small fraction of the sunlight spectrum ranging from 280 to 315 nm. Although this part of the spectrum represents only 1.5% of the light reaching the surface of the Earth, it can have multiple impacts on the life systems due to its high energy [3].

In plants, UV-B can induce detrimental or beneficial effects by the activation of UV-B-specific and/or -nonspecific signaling pathways depending on the energy level, exposure time, acclimation, peak wavelength, plant species, varieties and growth stages, and interaction with other environmental factors [4,5,6]. Previous studies have shown that high doses of UV-B can induce massive production of reactive oxygen species (ROS), which stimulates the production of hormones (i.e., jasmonic acid (JA) and salicylic acid (SA)) and triggers a general defense response [7,8]. High doses can also cause damage to DNA, cell membranes and proteins, and reduction of photosynthesis performance [9,10]. On the other hand, low and moderate doses of UV-B have been shown to stimulate photomorphogenic responses, which are often hormone-dependent (i.e., auxin (IAA)) linked to the UV-B RESISTANCE LOCUS 8 (UVR8) photoreceptor [2,11]. UVR8 action also stimulates the biosynthesis of protective secondary metabolites such as phenolic compounds (i.e., flavonoids) contributing to ROS detoxification [12,13]. Despite these rough categories of UV-B signaling (UV-B-specific (mediated by UVR8) and -nonspecific (general stress response)), it is possible that both pathways overlap at certain UV-B levels instead of being mutually exclusive [5].

Quinoa (*Chenopodium quinoa* Willd.) is a grain crop from the Andes and traditionally classified into five different groups based on its adaptation to specific environments: (i) ‘Highlands’ in Peru and Bolivia (>3500 m above the sea level (a.s.l.)); (ii) ‘Inter-Andean valleys’ in Colombia, Ecuador and Peru (2000 to 3500 m a.s.l.); (iii) ‘Salares’ in Bolivia, Chile and Argentina (salt flats); (iv) ‘Yungas’ in Bolivia (low altitude and humid valleys); and (v) ‘Sea level’ in Chile (low altitude) [14]. Quinoa is a very promising crop species due to its nutraceutical properties [15,16] and good tolerance to abiotic stress such as high salinity [17], drought [18] and high UV radiation [19]. For that reason, in the last twenty years, this crop has received worldwide interest and its cultivation has extended to Europe, Asia, North America and Africa [14]. In Europe, the quinoa breeding programme started in 1982 in Cambridge (United Kingdom) and then spread to other European countries. For instance, quinoa breeding in Denmark started in 1987 without registration of new varieties, while field trials in Finland began in 1997 [20]. Many studies have focused on salt and drought tolerance in quinoa, while those related to UV-B response are limited and still unclear [21]. A recent study showed that quinoa can regulate different mechanisms of response depending on the UV-B irradiation dosage [22]. Moreover, González et al. [19] demonstrated that quinoa varieties exhibit different morphological and physiological responses to solar UV-B. Therefore, motivated by these recent studies, the current work explores the effects of different UV-B dosages on hormonal regulation linked to plant morphology and defense, and whether these effects differ or are conserved among diverse quinoa varieties. In detail, var. *real* (Real) belongs to the group ‘Salares’ from the Southern Altiplano of Bolivia, where seeds are capable of growing at an altitude of ≥3700 m a.s.l. and 150 mm of annual precipitation [20]. Var. *titicaca* (Titicaca) is a Danish-bred variety containing Chilean and Peruvian genetic material and is propagated in field under non-saline conditions [23]. Var. *minttumatilda* (Minttumatilda) breading information is scarce, but a study reported that it originated from Southwestern Finland [24]. Peruvian varieties include *negra collana* (Negra Collana), *pasankalla* (Pasankalla), and *salcedo* INIA (Salcedo INIA) from ‘Highland’ that can tolerate cold and drought conditions, while *blanca* (Blanca) from ‘Inter-Andean valleys’ is susceptible to cold and is moderately drought tolerant [20]. Plants were irradiated with specific UV-B dosages [22] and changes in fluorescence parameters of photosystem II (PSII), flavonoids and hormones (IAA, JA, abscisic acid (ABA) and SA) were surveyed. The results should contribute information regarding UV-B response mechanisms in quinoa at certain UV-B levels, which may reflect variety-dependent constitutive and/or inducible strategies.

## 2. Materials and Methods

### 2.1. Plant Material and Growth Conditions

In this work, seven varieties of quinoa (*Chenopodium quinoa* Willd.) were used (Table 1). Seeds were surface-sterilized and germinated on MS medium as previously described [25]. After 9 days (d), seedlings were transferred to commercial soil and grown in growth chambers under a 12 h light/12 h dark photoperiod, temperature 22 ± 1 °C, 75% relative humidity and 100 μmol m^−2^ s^−1^ photosynthetically active radiation (PAR). Pots were irrigated with distilled water three times a week and with nutrient solution (NPK 5-5-5) once a week.

### 2.2. UV-B Radiation Treatments

At 33 d after sowing, quinoa plants were subjected to UV-B treatment as described by Huarancca Reyes et al. [26]. Briefly, chambers were kept at a 12 h light/12 h dark photoperiod, temperature 22 ± 1 °C, 75% relative humidity and 100 μmol m^−2^ s^−1^ PAR. Exposure to UV-B was applied from three Philips TL 20W/01RS UV-B Narrowband lamps (Koninklijke Philips Electronics, Eindhoven, The Netherlands) with peak emission at 311 nm. The applied UV-B corresponded to 3.04 or 6.08 kJ m^−2^ d^−1^ biologically effective radiation and the irradiation was performed in the middle of the light period for 3 d. At the end of the experiment, three groups of plants were obtained depending on treatment: 0 (referred to as control), 9.12 and 18.24 kJ m^−2^ UV-B. It should be mentioned that the period of UV-B irradiance (3 d of treatment) used in this work was selected according to a previous study [22], where quinoa plants develop UV-B-specific or-independent pathways depending on the UV-B irradiation dosage.

### 2.3. Chlorophyll Fluorescence

Chlorophyll fluorescence was measured on control and UV-B treated plants using a pulse amplitude-modulated fluorometer (Mini-PAM; Heinz Walz GmbH, Effeltrich, Germany). The operational quantum efficiency of photosystem II (PSII) photochemistry in the light (Φ_PSII_) was determined under actinic illumination of 100 μmol m^−2^ s^−1^ as Φ_PSII_ = (*F_m_*′ – *F*′)/*F_m_*′, where *F_m_*′ represents the maximum fluorescence yield emitted by the leaves after superimposing a saturating light flash during actinic light exposition, and *F*′ represents the fluorescence yield emitted under actinic illumination. Plants were pre-darkened for 30 min and the potential efficiency of PSII photochemistry was evaluated as *F_v_*/*F_m_* = (*F_m_* – *F_o_*)/*F_m_*, where *F_o_* and *F_m_* are the minimum and the maximum fluorescence yield emitted by the leaves in the dark-adapted state, respectively [27,28]. All measurements included three biological replicates.

### 2.4. Flavonoid Extraction and Determination

Leaf samples were extracted as previously described [22]. Total flavonoids were determined referring to Kim et al. [29] by measuring the absorbance at 510 nm. Three biological replicates were considered for this analysis, and results were reported as mg catechin equivalents g^−1^ of fresh weight (FW).

### 2.5. Hormone Extraction and Quantification

Approximately 500 mg of leaves from control and UV-B treated plants were collected. The material was homogenized in cold 80% (*v*/*v*) methanol (1:5, *w*/*v*) using a microdevice as reported in Mannucci et al. [30]. Deuterated [^2^H_4_]-SA, [^2^H_5_]-JA, [^2^H_6_]-ABA (CDN Isotopes Inc., Pointe-Claire, QC, Canada) and [^13^C_6_]-IAA (Cambridge Isotopes Laboratories Inc., Andover, MA, USA) were added as internal standards to account for purification losses. Methanol was evaporated under a vacuum at 35 °C and the aqueous phase was partitioned against ethyl acetate, after adjusting the pH to 2.8. The extracts were dried and resuspended in 0.3–0.5 mL of water with 0.01% acetic acid and 10% methanol. HPLC analysis was performed with a Kontron instrument (Munich, Germany) equipped with a UV absorbance detector operating at 214 nm. The samples applied to an ODS Hypersil column (150 × 4.6 mm I.D. and 5 μm of a particle size) (Thermo Fisher Scientific, USA), were eluted at a flow rate of 1 mL min^−1^. The column held constant at 10% methanol for 5 min of the run, followed by a double gradient elution from 10% to 30% and 30% to 100% over 20 min. The fraction corresponding to the elution volume of SA and IAA was dried and silylated with N,O-bis(trimethylsilyl) trifluoroacetamide containing 1% trimethylchlorosilane (Pierce, Rockford, IL, USA) at 70 °C for 1 h, while the fraction corresponding to the elution volume of ABA and JA was dried under vacuum, and methylated with ethereal diazomethane. Chromatography-tandem mass spectrometry (GC-MS/MS) analysis was performed on a Saturn 2200 quadrupole ion trap mass spectrometer coupled to a CP-3800 gas chromatograph (Varian Analytical Instruments, Walnut Creek, CA, USA) equipped with a MEGA 1MS capillary column (30 m × 0.25 mm I.D. and 0.25 µm film thickness) (Mega, Milano, Italy). The carrier gas was helium, which was dried and air free, with a linear speed of 60 cm s^−1^. The oven temperature was maintained at 80 °C for 2 min and increased to 300 °C at a rate of 10 °C min^−1^. The injector and transfer line were set at 250 °C and the ion source temperature at 200 °C. Full scan mass spectra were obtained in EI+ mode with an emission current of 10 µA and an axial modulation of 4 V. Data acquisitions was from 100 to 600 Da at a speed of 1.4 scan s^−1^. Final data were the means of three biological replicates. Hormones were identified by comparison of full mass spectra with those of authentic compounds. Quantification was carried out by reference to standard plots of concentration ratios versus ion ratios, obtained by analyzing known mixtures of unlabeled and labelled hormones. Three biological replicates were considered and results were expressed as ng^−1^ of FW.

### 2.6. Statistical Analysis

Values presented are means ± standard error of three replicates. After performing Bartlett’s test for homogeneity of variance, data were subjected to one-way analysis of variance (ANOVA) with treatment (control, 9.12 and 18.24 kJ m^−2^ UV-B) as main factor. Significant differences among means were estimated at the level of *P* < 0.05 by Tukey’s test, using STATISTICA for Windows version 13.4.0 (Start-Soft, Inc., Tulsa, OK, USA).

Flavonoids, hormones and photosynthesis-related parameters (each UV-B treatment with respect to the control) corresponding to seven quinoa varieties were classified according to Ward’s method of hierarchical clustering using software SPSS version 25.0 (SPSS, Inc., Chicago, IL, USA). Cluster intervals were set via Squared Euclidean distance. For Principal Component Analysis (PCA), datasets (same as for clustering) were scaled (1/squared root (SD)) and mean-centered (variables mean) prior to modelling. The latter was done using the SIMCA software package (SIMCA version 15.0, Umetrics AB, Umea, Sweden).

## 3. Results

**Figure 1:**
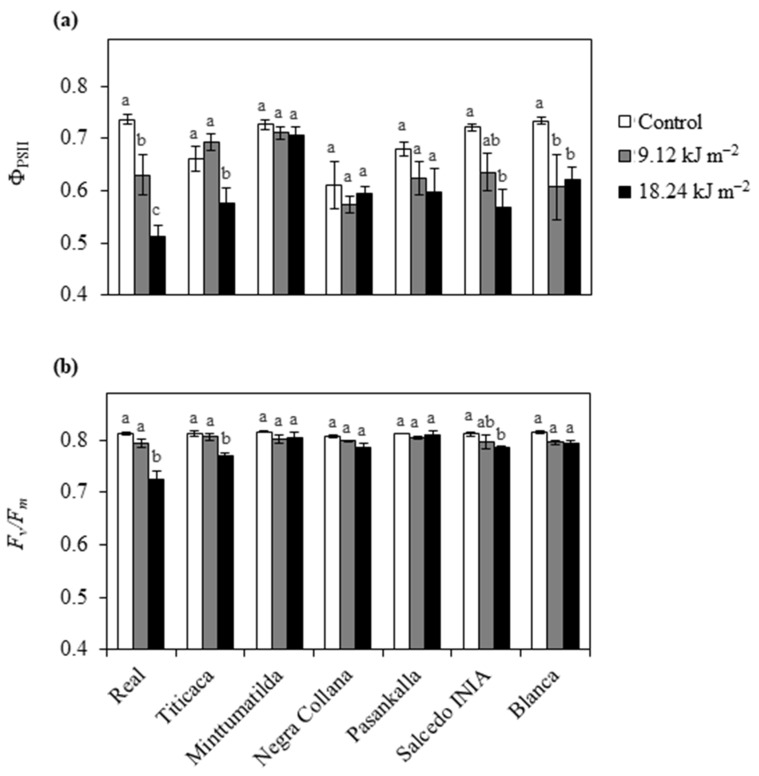
Effects of UV-B on the actual (Φ_PSII_) and maximum (*F_v_*/*F_m_*) efficiency of PSII photochemistry. Different quinoa varieties were exposed to 0 (Control), 9.12 and 18.24 kJ m^−2^ UV-B. Parameters of Φ_PSII_ (**a**) and *F_v_*/F_m_ (**b**) were evaluated in leaves at 3 days of treatment. Error bars represent the standard error of the mean (n = 3). Different letters indicate significant differences between means within the same variety (*P* < 0.05, Tukey’s test).

Photosynthetic-related parameters are shown in Figure 1, which include the actual photon yield of PSII photochemistry (Φ_PSII_) (Figure 1a) and the maximal PSII photochemical efficiency (*F_v_*/*F_m_*) (Figure 1b). Real showed a gradual and significant decrease of Φ_PSII_ with the increase of UV-B irradiation dosage; a similar pattern was followed by Salcedo INIA but a significant decline was only observed in plants exposed to 18.24 kJ m^−2^ UV-B in comparison with the control (Figure 1a). Titicaca showed a steep decline of Φ_PSII_ only when plants were exposed to 18.24 kJ m^−2^ UV-B, while Blanca experienced this decline even at half the maximum UV-B dose (9.12 kJ m^−2^) (Figure 1a). Conversely, the Φ_PSII_ values in Minttumatilda, Negra Collana and Pasankalla exposed to both UV-B doses (9.12 and 18.24 kJ m^−2^) showed no significant differences with respect to their controls (Figure 1a). Evaluation of *F_v_*/*F_m_* in UV-B exposed plants showed that UV-B negatively affected the varieties Real, Titicaca and Salcedo INIA only when the dose was 18.24 kJ m^−2^ (Figure 1b). Regarding the other varieties, no effects on *F_v_*/*F_m_* were observed with either 9.12 or 18.24 kJ m^−2^ UV-B compared to their controls (Figure 1b).

**Figure 2:**
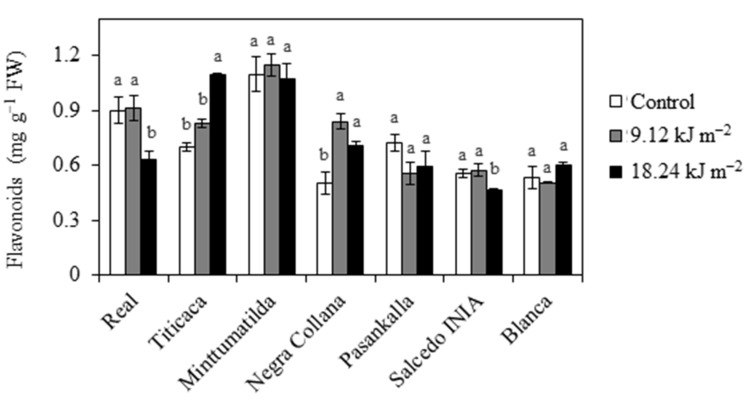
Effects of UV-B on flavonoids concentration. Different quinoa varieties were exposed to 0 (Control), 9.12 and 18.24 kJ m^−2^ UV-B. Flavonoids (mg catechin equivalents g^−1^ FW) were evaluated in leaves at 3 days of treatment. Error bars represent the standard error of the mean (n = 3). Different letters indicate significant differences between means within the same variety (*P* < 0.05, Tukey’s test). FW, fresh weight.

Total flavonoid concentration was not affected by UV-B irradiation in Minttumatilda, Pasankalla and Blanca, while the other varieties showed some effects depending on the UV-B dosage (Figure 2). Real and Salcedo INIA shared a common reduction of flavonoids only after receiving the maximum UV-B dose (18.24 kJ m^−2^) (Figure 2). Titicaca gradually increased flavonoid concentration with the increase of UV-B dose, but the only significant difference was observed with 18.24 kJ m^−2^ (Figure 2). A different pattern was observed in Negra Collana, which significantly increased flavonoids with 9.12 kJ m^−2^ UV-B and this level remained relatively stable when the UV-B dose was duplicated (Figure 2).

**Figure 3:**
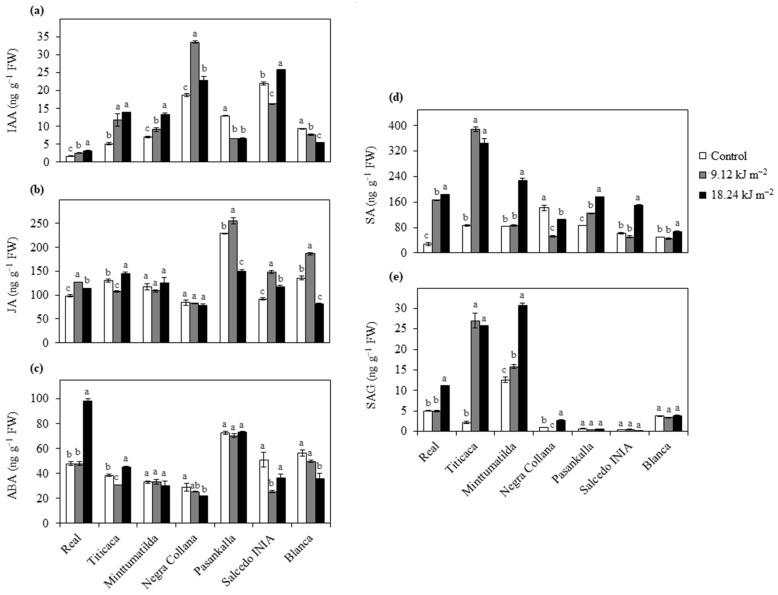
Effects of UV-B on plant growth regulators. Different quinoa varieties were exposed to 0 (Control), 9.12 and 18.24 kJ m^−2^ UV-B. Hormones (ng g^−1^ FW) including IAA (**a**), JA (**b**), ABA (**c**), SA (**d**) and SAG (**e**) were evaluated in leaves at 3 days of treatment. Error bars represent the standard error of the mean (n = 3). Different letters indicate significant differences between means within the same variety (*P* < 0.05, Tukey’s test). IAA, indoleacetic acid. JA, jasmonic acid. ABA, abscisic acid. SA, salicylic acid. SAG, salicylic acid glucoside. FW, fresh weight.

To assess the effect of acute UV-B on hormone regulation in different quinoa varieties, IAA, JA, ABA, SA and SAG were investigated (Figure 3). Real and Minttumatilda showed a gradual and significant increase of IAA in response to the UV-B dose, while Negra Collana and Titicaca reached the maximum IAA level with 9.12 kJ m^−2^ UV-B followed by a significant decrease or remained unchanged when the UV-B dose was duplicated, respectively (Figure 3a). In contrast, Blanca showed a steady and significant decline of IAA with increasing UV-B dose, whereas Pasankalla exposed to 9.12 kJ m^−2^ UV-B displayed a sharp decline of IAA maintaining the same level by increasing the dose (Figure 3a). Salcedo INIA showed the highest levels of IAA in their control leaves, which after UV-B irradiation presented a significant decrease or increase depending on the dosage (9.12 or 18.24 kJ m^−2^ respectively) (Figure 3a). In the case of JA, Real and Salcedo INIA showed similar patterns where control had lower JA levels than UV-B irradiated leaves, which differed according to the UV-B dose, as JA levels were higher in leaves irradiated with 9.12 than with 18.24 kJ m^−2^ (Figure 3b). Although Pasankalla and Blanca also presented higher JA in their leaves irradiated with 9.12 kJ m^−2^ in comparison with their controls, these levels were steeply reduced when leaves were irradiated with 18.24 kJ m^−2^ UV-B (Figure 3b). In contrast, Titicaca showed an opposite JA pattern with respect to Pasankalla and Blanca, while Minttumatilda and Negra Collana did not show significant differences between the JA levels in their UV-B irradiated and control leaves (Figure 3b).

Analysis of ABA resulted in four different patterns depending on the UV-B dose (Figure 3c). Real showed a steep increase of ABA after receiving 18.24 kJ m^−2^ UV-B in comparison with the control and 9.12 kJ m^−2^ irradiated leaves that shared the same levels (Figure 3c). Titicaca and Salcedo INIA significantly decreased their ABA concentrations in 9.12 kJ m^−2^ irradiated leaves with respect to their controls, followed by a significant increase when the UV-B dose was duplicated, reaching higher levels than that of the control in Titicaca or the same levels as the control in Salcedo INIA (Figure 3c). The concentration of ABA in Negra Collana and Blanca gradually decreased with increasing UV-B dose, while in Minttumatilda and Pasankalla, it did not show significant differences between control and UV-B irradiated leaves (Figure 3c).

The levels of SA in Real, Titicaca and Pasankalla were higher in UV-B treated leaves than controls with some differences; while Real and Pasankalla showed an increase of SA with extended dose, Titicaca did not show differences between UV-B doses (Figure 3d). Minttumatilda, Salcedo INIA and Blanca did not show differences in SA between control and 9.12 kJ m^−2^ irradiated leaves, but these levels were significantly increased when the UV-B dose was duplicated (Figure 3d). On the other hand, UV-B significantly reduced the SA in Negra Collana with respect to the control, where this decline was more pronounced in 9.12 than in 18.24 kJ m^−2^ treated plants (Figure 3d). Regarding SAG determination, Pasankalla, Salcedo INIA and Blanca did not show differences in SAG levels between control and UV-B treatment, while the SAG patterns in Titicaca and Negra Collana were similar to that of SA (Figure 3e). In the case of Minttumatilda, a gradual increase of SAG was observed with increasing UV-B dose, whereas Real showed a significant increase only when plants were irradiated with 18.24 kJ m^−2^ UV-B (Figure 3e).

**Figure 4:**
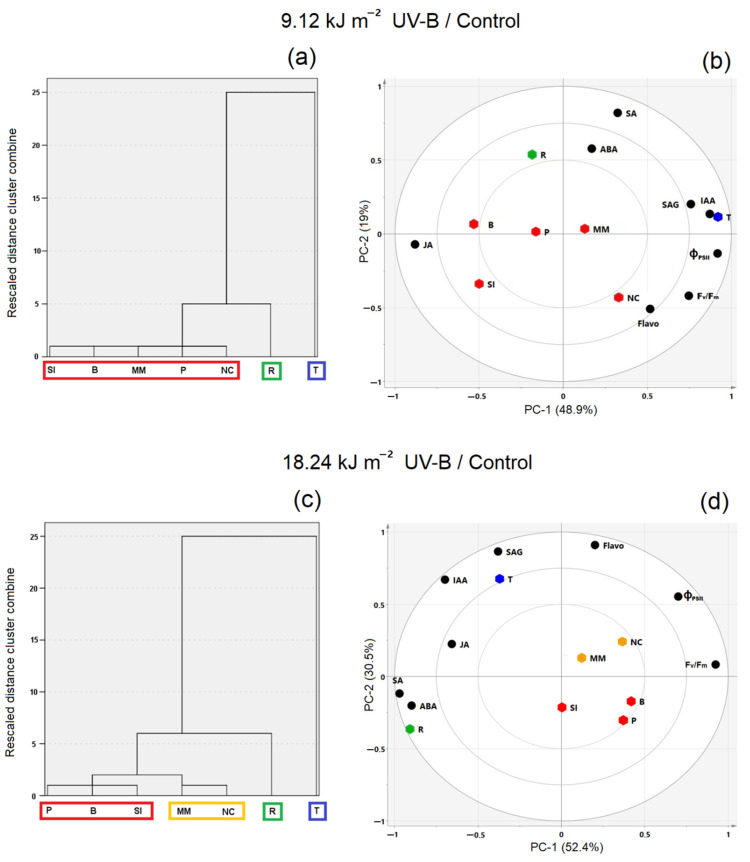
Dendrogram and Principal Component Analysis (PCA; percentage of explained variance of the models, 67.9 (**b**) and 82.9% (**d**)) plots corresponding to seven quinoa varieties exposed to 9.12 and 18.24 kJ m^−2^ UV-B (**a**,**b** and **c**,**d**, respectively). Clusters in dendrogram were color-linked to scores in PCA. Circles indicate 50 and 100% limits for the explained variance of the PCA models. Scores: Quinoa variety *real* (R), *titicaca* (T), *minttumatilda* (MM), *negra collana* (NC), *pasankalla* (P), *salcedo* INIA (SI) and *blanca* (B). Loadings: Photosynthesis-related parameters (*F_v_*/*F_m_* and Φ_PSII_), flavonoids (Flavo) and hormones (IAA, SA, SAG, JA and ABA).

After exposure to 9.12 kJ m^−2^ UV-B, three clusters were clearly identified (Figure 4a): Titicaca (i); Real (ii); Negra Collana, Pasankalla, Minttumatilda, Blanca, Salcedo INIA (iii). High values of IAA, SAG, Φ_PSII_ and *F_v_*/*F_m_* were observed in Titicaca, while ABA and SA were mostly observed in Real (Figure 4b). Despite unremarkable differences, some samples showed minor tendencies towards JA (i.e., Salcedo INIA and Blanca) or flavonoids (i.e., Negra Collana) (Figure 4b). After exposure to 18.24 kJ m^−2^ UV-B, four clusters were identified (Figure 4c): Titicaca (i); Real (ii); Negra Collana, Minttumatilda (iii); Salcedo INIA, Blanca, Pasankalla (iv). Titicaca was observed to be high in IAA, SAG and flavonoids, while Real was high in SA and ABA (Figure 4d). JA was moderately associated with Titicaca and Real (Figure 4d). From the remaining samples, Minttumatilda and Negra Collana showed a slight tendency towards Φ_PSII_, *F_v_*/*F_m_* and flavonoids, and the rest of the samples showed less noticeable differences (Figure 4d).

## 4. Discussion

PSII functionality is commonly accepted as an indicator of stress status in plants. Here, Real exposed to 9.12 kJ m^−2^ UV-B was capable of maintaining *F_v_*/*F_m_* in the optimal range, while 18.24 kJ m^−2^ causes a strong decrease of *F_v_*/*F_m_* that is typical of stressed plants [31]. It has been demonstrated that UV-B induces ROS (i.e., H_2_O_2_) production in chloroplasts, interfering with the light reaction process, and depending of the level and duration of UV-B exposure, the photosynthetic performance of the plant can be compromised [32]. Thus, according to the effect of UV-B doses on the PSII functionality, it is possible that 18.24 kJ m^−2^ overtakes the threshold and becomes detrimental causing ‘distress’, while 9.12 kJ m^−2^ causes ‘eustress’ in Real [8]. Titicaca showed similar photosynthesis performance to Real in response to both UV-B doses, while in Minttumatilda no effects were reported in response to 9.12 and 18.24 kJ m^−2^ UV-B. The different UV-B sensitivity in both European varieties may be linked to the parental lines and the time when breeding started [20,23,24]. Interestingly, one parental line of Titicaca is from southern Chile where salinity is predominant as Real’s natural environment; thus, it is possible that both varieties have a similar response at the photosynthetic level and sense 9.12 kJ m^−2^ as ‘eustress’, whereas they sense 18.24 kJ m^−2^ as ‘distress’. In contrast, Minttumatilda may perceive both UV-B doses as ‘eustress’, as its genome probably did not change significantly over the years in comparison with Titicaca. Among Peruvian varieties, only Salcedo INIA showed a permanent damage of the PSII when exposed to 18.24 kJ m^−2^ UV-B, suggesting that this dose may represent a ‘distress’ for this variety. Moreover, Salcedo INIA showed similar UV-B responses to Real, at least at the photosynthetic level, most probably due to the common ‘Salares’ background [33]. In contrast, the other Peruvian varieties did not show damage in their PSII in response to UV-B, indicating that both doses used in this study did not overtake their detrimental thresholds.

Flavonoids are secondary metabolites with UV screening and antioxidant properties [34]. In Real, 9.12 kJ m^−2^ did not induce changes in the concentration of flavonoids, suggesting that these UV-absorbing pigments might be less important for UV-B sensing at least under this condition. A similar result occurred in leaves of Cristalina variety naturally adapted to high UV locations (3500–4100 m a.s.l.) [19]. However, changes in the concentration of individual flavonoids in response to this UV-B dose cannot be excluded [35]. In contrast, 18.24 kJ m^−2^ caused a drop in flavonoids, which is probably linked to the severe ROS overproduction that can change the redox balance towards oxidative stress, causing the continuous oxidation of these metabolites [22]. The possibility that a high UV-B dose induces flavonoid transport from shoot to roots for specific signaling cannot be ruled out [36,37]. In Titicaca, these metabolites increased with UV-B doses, suggesting that they might be important for the UV-B sensing in this variety. Differently to Real, Titicaca was propagated in low UV-B locations and this could be the reason why flavonoids increase in response to both UV-B doses as occurs in lowland varieties such as var. *faro roja* [38]. Minttumatilda has the highest level of flavonoids under control conditions and this level did not change in response to UV-B, suggesting that its high basal level is a constitutive strategy to overcome this stress. However, since there is a tradeoff between photosynthetic and non-photosynthetic pigments in response to UV-B [39], it could be interesting to evaluate these pigments and their photoprotection role in Minttumatilda. The level of flavonoids in response to UV-B differed between Peruvian varieties. Salcedo INIA showed a similar pattern to Real, probably related to the common background [33]; thus, 9.12 kJ m^−2^ may be a dose where flavonoids are not important for UV-B sensing, while 18.24 kJ m^−2^ may change the redox balance towards oxidative stress and cause the continuous oxidation of these metabolites [40]. Although Negra Collana and Pasankalla were collected from local cultivars in similar locations [33], the foremost showed an induction of flavonoids whereas the latter did not show changes in response to UV-B independently of dose. Moreover, similar to Pasankalla, Blanca did not change its flavonoid levels in response to UV-B even though the varieties are grown in different ecoregions. Thus, similar to salinity tolerance, UV-B responses in quinoa do not necessarily correlate with its geographical distribution [41].

Hormonal analysis in Real and both European varieties revealed that IAA content increased with UV-B. Ringli et al. [42] found that a modified flavonoid profile induces changes in auxin concentration resulting in alterations in *Arabidopsis* plant morphology (i.e., leaf curling). Moreover, Neugart et al. [43] found that flavonoid profile changes depending on the UV-B dose in kale. Thus, this suggests that in these varieties UV-B may change the profile of flavonoids that in turn induce IAA synthesis; however, a detailed flavonoid analysis is needed to confirm this hypothesis. In contrast, Pasankalla and Blanca reduced IAA content in response to UV-B. It has been demonstrated that peroxidases are capable of oxidizing many phenolics, contributing to the PSII protection from UV radiation as well as controlling leaf and plant architecture by the oxidation of IAA [44]. Moreover, quinoa seedlings exposed to UV-B showed an increase of the activity of a specific peroxidase associated with lignification processes, resulting in an effective mechanism against UV-B [45]. Taken together, this suggests that UV-B tolerance in Pasankalla and Blanca is linked to IAA degradation associated with the increased activity of a specific peroxidase; however, more studies are needed to confirm this hypothesis. Negra Collana and Salcedo INIA showed both an increase and reduction in IAA content depending on the UV-B dose, probably due to changes in the flavonoids profile [42,43] and/or activation of specific peroxidases [44].

ABA is mainly known to play an important role in drought and high salinity, but its accumulation is also related to UV-B stress [2]. Real only showed an accumulation of ABA when exposed to 18.24 kJ m^−2^. It is known that stomatal movement triggered by UV-B can result from the crosstalk between ABA- and UVR8-dependent pathways [46]. Moreover, both doses used in this study led to the reduction of stomatal conductance due to stomatal closure in this variety [22]. Therefore, it seems that Real perceives 18.24 kJ m^−2^ as ‘distress’, inducing a general stress response similar to other ‘Salares’ varieties facing salt stress [47], while 9.12 kJ m^−2^ is perceived as ‘eustress’. In contrast, Titicaca may sense UV-B as ‘eustress’ (9.12 kJ m^−2^) or ‘distress’ (18.24 kJ m^−2^) to different degrees in comparison with Real, which is reflected in its lower ABA accumulation, which may be an antagonist effect of flavonoids [48]. On the other hand, ABA content in Minttumatilda did not change in response to both UV-B doses, which may be a result of the unchanged accumulation of flavonoids [48]. Peruvian varieties showed unaltered or reduced ABA content in response to UV-B doses, indicating that they are quite tolerant to UV-B. The continuous reduction of ABA level in Negra Collana exposed to UV-B may be a result of the antagonist effect of flavonoids on ABA [48], while the unchanged ABA content in Pasankalla under both UV-B doses may be linked to the unaffected flavonoids level. In the case of Salcedo INIA and Blanca, both varieties showed low ABA levels in response to UV-B, together with the fact that their flavonoids are not increased. This suggests that ABA-independent mechanisms are committed in response to UV-B, as occurred in other varieties when faced drought stress [49]. The effect of other hormones such as cytokinin and ethylene on ABA cannot be ruled out.

JA and SA are hormones involved in the biotic stress response, and thus, are typically associated with high UV-B stress levels [2]. However, it is possible that certain UV-B levels induce defense signaling pathways without an evident ‘distress’ [5]. For instance, low to moderate UV-B doses can increase defense metabolite content together with SA- and JA-related gene transcription in broccoli sprouts [50], while elevated UV-B in two cultivars of pea and mug bean caused SA and JA accumulation to different extents, where SA is associated with UV-B sensitivity and JA with UV-B tolerance [51,52]. Here, Real increased JA as well as SA content when exposed to both UV-B doses; however, JA was to a much lower degree than SA, indicating that this variety may be susceptible to 9.12 and 18.24 kJ m^−2^ UV-B but to different extents. Concordantly, 18.24 kJ m^−2^ strongly increased not only SA but also its glucoside form (SAG), whose reversible conversion (SA_active_↔SAG_inactive_) is a mechanism activated by plants to prevent damage [53], suggesting that Real senses this UV-B dose as ‘distress’, increasing the pool of SA (active and conjugated forms) much more. Similarly, European varieties seem to have some grade of sensitivity to UV-B. Titicaca showed the strongest accumulation of SA pool independently of UV-B dose, reflecting a very efficient reaction to this stress, which may be related to its adaptation to low UV-B conditions during its breeding program. In contrast, Minttumatilda increased the SA pool only after exposure to 18.24 kJ m^−2^ UV-B. This, together with the fact that both UV-B doses used in this study did not affect its PSII functionality, lead us to hypothesize that 18.24 kJ m^−2^ may induce a defense response without ‘distress’ in Minttumatilda. Concerning Peruvian varieties, Pasankalla, Salcedo INIA and Blanca exposed to 9.12 kJ m^−2^ accumulate JA while SA content was slightly increased or unaffected, indicating that these varieties are quite tolerant to this UV-B dose. On the other hand, 18.24 kJ m^−2^ induced the reduction of JA and an increase in SA content, indicating that this dose may activate some stress-related responses. Interestingly, the SA accumulation extent in Pasankalla, Salcedo INIA and Blanca was much lower than in Real and Titicaca, suggesting that these Peruvian varieties are more resistant to 18.24 kJ m^−2^ UV-B. In the case of Negra Collana, UV-B did not change JA levels but it reduced SA content, indicating that both UV-B doses were sensed as ‘eustress’.

## 5. Conclusions

Quinoa is intrinsically well adapted to high UV-B as it is native from high altitude locations. Here, under controlled conditions, we show that the sensitivity to short acute UV-B doses in varieties from different habitats are influenced by their parental lines and breeding time in a new environment. In fact, Titicaca with a longer breeding program than Minttumatilda showed a defined UV-B response, forming a unique cluster separated from the other varieties used in this study, while Minttumatilda shared some similarities with Peruvian varieties in response to UV-B. Moreover, UV-B sensitivity does not necessarily correlate with quinoa’s geographical distribution. Indeed, Real was more susceptible to acute UV-B than all Peruvian varieties, even though the ‘Salares’ quinoa can tolerate the most severe natural conditions. This fact suggests that the crosstalk between abiotic stresses is likely to be advantageous for plants. Moreover, all Peruvian varieties used in this study were quite tolerant to 9.12 kJ m^−2^ UV-B and differences in sensitivity were observed when increasing the dose.

This study points out that the role of flavonoids in UV-B response seems to be different depending on variety. This study tries to establish a criterion for describing the UV-B sensitivity in quinoa varieties depending on their hormonal regulation. Overall, we found that the extent of changes in JA and SA correlate with UV-B tolerance, while the increase of ABA was mainly related to UV-B stress under controlled conditions.

## Figures and Tables

**Table 1:** List of the quinoa varieties used in this study. All seeds were harvested in 2017 and were obtained from different cultivation areas with the exception of *real*, which was commercially obtained.

Variety Name	Cultivation Area		
	Country	Latitute, Longitude	Elevation ^b^
*real*	Seeds purchased from Priméal (Peaugres, France)		
*titicaca*	Denmark	55.67, 12.30	35 m a.s.l.
*minttumatilda* ^a^	Finland	60.55, 22.70	33 m a.s.l.
*negra collana*	Peru	−16.32, −69.23	3870 m a.s.l.
*pasankalla*	Peru	−16.32, −69.23	3870 m a.s.l.
*salcedo* INIA	Peru	−15.88, −70.00	3843 m a.s.l.
*blanca*	Peru	−13.07, −74.38	3216 m a.s.l.

^a^ Population variety. ^b^ a.s.l., above the sea level.

## Data Availability

Data is contained within the article.
